# Crosstalk Analysis of a CMOS Single Membrane Thermopile Detector Array

**DOI:** 10.3390/s20092573

**Published:** 2020-04-30

**Authors:** Ying Dai, Syed Zeeshan Ali, Richard Hopper, Claudio Falco, Prakash Pandey, Chris Oxley, Daniel Popa, Florin Udrea

**Affiliations:** 1 Department of Engineering, University of Cambridge, Cambridge CB3 0FA, UK; rhh39@cam.ac.uk (R.H.); dp387@cam.ac.uk (D.P.); fu10000@cam.ac.uk (F.U.); 2 Flusso Limited, Cambridge CB4 0DL, UK; zeeshan.ali@flussoltd.com (S.Z.A.); claudio.falco@flussoltd.com (C.F.); 3 Faculty of Computing, Engineering and Media, De Montfort University, Leicester LE1 9BH, UK; prakash.pandey@dmu.ac.uk (P.P.); choxley0@gmail.com (C.O.)

**Keywords:** infrared sensor, thermopile, CMOS, MEMS, seebeck effect, crosstalk

## Abstract

We present a new experimental technique to characterise the crosstalk of a thermopile-based thermal imager, based on bi-directional electrical heating of thermopile elements. The new technique provides a significantly simpler and more reliable method to determine the crosstalk, compared to a more complex experimental setup with a laser source. The technique is used to characterise a novel single-chip array, fabricated on a single dielectric membrane. We propose a theoretical model to simulate the crosstalk, which shows good agreement with the experimental results. Our results allow a better understanding of the thermal effects in these devices, which are at the center of a rising market of industrial and consumer applications.

## 1. Introduction

Low-cost and low-power consumption thermal infrared (IR) focal plane arrays (FPAs) are increasingly important for many applications [1,2,3,4]. These consist of two-dimensional arrays of imaging pixels (typically bolometers [5], thermopiles [6] or pyroelectric detectors [7]), found within the Internet of Things (IoT) environment [8], and/or consumer electronics [9] applications, including, e.g., activity recognition for care services [10] and presence detection for security [11]; applications which require high volume device manufacturability and battery powered operation [9]. Currently, bolometers [1,5,12] and thermopiles [5,6,13,14,15] dominate the low-cost IR-FPA market [4]. Bolometers detect temperature induced changes in resistance (*R*), created when the detector element is heated by incident IR radiation. A voltage signal ($V_{B}$) is obtained by providing a suitable biasing current to the detector element [16]. Compared to bolometers, which often require specialist fabrication and packaging [1,12], IR-FPAs based on thermopiles are easier to manufacture using standard low-cost Complementary Metal–Oxide–Semiconductor (CMOS) fabrication processes [13,14]. In addition, they detect temperature induced changes in electromotive force (i.e., the thermoelectric voltage ($V_{T}$) generated due to the Seebeck effect [17]), and thus do not require a biasing current, thereby simplifying the interface circuitry [13,14].

Thermopiles are typically made of a series of thermocouples [18], each comprising two conductors (e.g., p${}^{+}$/n${}^{+}$ doped polysilicon [14,19,20], or silicon [13,20]) with dissimilar Seebeck coefficients (typically ∼300 μV/K [20]), where one “hot junction” can be heated while the opposite “cold junction” is thermally bonded to a heat-sinking substrate [21]. The thermocouple generates a voltage $V_{T}=\alpha \Delta T$ [18] when a temperature gradient ΔT is present across its junctions, where α is the Seebeck coefficient (a measure of the material’s efficiency to thermally generate a voltage [17]). Thermopile-based FPAs are composed of an array of thermopile-based pixels located at the focal plane of a lens [4], and produce a thermal image by detecting ΔTs between pixel elements. The hot junctions of each pixel’s thermocouples are typically thermally isolated by a thin dielectric membrane [21,22]. A voltage $V_{T}=N\alpha \Delta T$ (proportional to the number of thermocouples *N*), is generated when the membrane is IR irradiated [18]. We recently demonstrated a low-cost (<1$) and low-power-consumption (∼mW) CMOS-based thermopile FPA fabricated on a single dielectric membrane, employing standard CMOS tungsten (W) metallization layers for heat-sinking of the thermopile cold junctions, and exemplified its potential by applying it to gesture recognition and people-counting [13].

An important figure of merit of a FPA is the pixel-to-pixel crosstalk (*C*) (i.e., the unwanted transfer of signals between pixels [23]), which can affect the spatial resolution of the detector and thus complicate the reconstruction of the desired image [24]. For a thermopile array, *C* is defined as the ratio between the $V_{T}$ signal generated by an optically irradiated pixel ($V_{T1}$) and that of an adjacent non-irradiated pixel ($V_{T2}$), i.e., $C=V_{T2}/V_{T1}$ [6]. In thermal FPAs, *C* is dependent on inter-pixel heat diffusion [6], and is typically measured by optically irradiating (e.g., by a laser source [6]) a pixel and comparing its $V_{T}$ signal to that of adjacent pixels [6,19]. However, imperfections in the laser focusing can lead to optical leakage from the laser spot, which can be challenging to control and/or quantify [6,19]. A different approach for *C*-measurement is to use an on-chip heater for thermal excitation [6]. However, this additional structure increases the fabrication complexity and may compromise the thermal performance of the device.

Here we introduce a novel approach for measuring the crosstalk of a thermopile-based FPA without using a laser source, or an on-chip heater. Our approach uses bi-directional electrical biasing of the thermopile elements themselves to obtain the thermoelectric voltages needed for crosstalk calculations. Moreover, we propose a numerical model to simulate the crosstalk of a single membrane thermopile array design, which we use to better understand the crosstalk formation of these devices.

## 2. Device Fabrication

**Figure 1:**
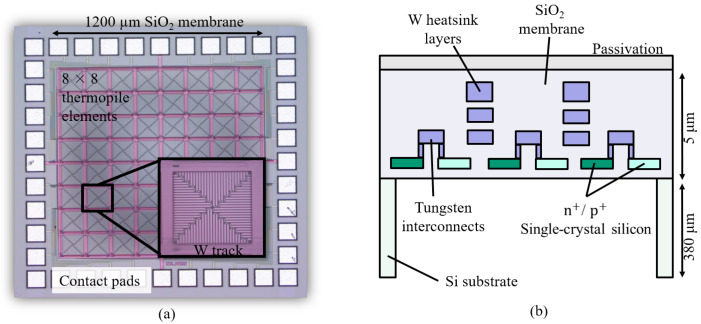
(**a**) Optical image of the thermopile array with a magnified image (colour-shifted) of an individual pixel. Chip size = 1.76 mm × 1.76 mm. (**b**) Cross-sectional view of the numerical model (not to scale) showing the single-crystal Si $p^{+}$/$n^{+}$ elements and tungsten (W) layers of the thermopile array.

The thermopile detector array is fabricated on a single membrane using a commercial 1 μm silicon on insulator (SOI)-CMOS process on 6 inch Si wafers. The pixels are formed by highly doped $p^{+}$ and $n^{+}$ single-crystal Si layers formed within the SOI layer. The interconnection between the $p^{+}$ and $n^{+}$ elements, and the heatsinking tracks between the pixels are formed by three W interconnect layers, with 20 μm track widths, and ∼1 μm total thickness. W is an interconnect metal which can be employed in standard high-temperature CMOS processes [25] and it is implemented for heatsinking due to its much higher thermal conductivity (∼80 W/mK) when compared to that of silicon dioxide (SiO${}_{2}$, ∼0.8 W/mK) which forms the membrane of our device. Figure 1a shows the optical image of our device, with the inset showing the zoomed-in structure of a single pixel. The SiO${}_{2}$ membrane is ∼5 μm thick and has an area of 1200 μm × 1200 μm (150 μm × 150 μm for a single pixel), see Figure 1b. The layers are grown on a 380 μm thick Si substrate, back-etched using deep reactive ion etching (DRIE) to form the membrane, with the first SiO${}_{2}$ layer acting as an etch stop. A device, with array sizes of 8×8 pixels, is fabricated as a proof-of-concept. Each pixel consists of a thermopile with 52 thermocouple pairs. The thermopiles have their cold junctions placed adjacent to the surrounding heatsinking tracks, formed by the three W layers.

## 3. Experimental Results

**Figure 2:**
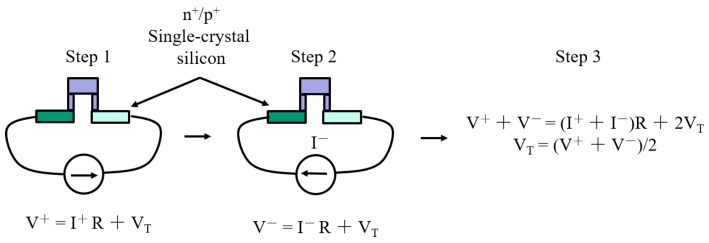
Schematic diagram showing the bi-directional electrical biasing measurement method.

To evaluate the crosstalk, we use a bi-directional electrical biasing approach to obtain the voltage $V_{T}$ generated by a thermopile (pixel) under thermal stress, as shown in Figure 2. The pixel is thermally stressed by Joule heating [26], i.e., by applying a range of biasing currents (from I∼ 10 μA to 200 μA in our case) to the thermopile elements, resulting in a heat load ($RI^{2}$) proportional to the track resistance (*R*). This gives rise to a ΔT across its thermocouple’s junctions and thus a $V_{T}$. To extract $V_{T}$, we apply a current in both negative and positive directions, as shown in Figure 2. When the $p^{+}$ and $n^{+}$ Si elements of the pixel are *I*-biased, the total generated voltage will then contain an ohmic drop (V=IR) contribution, caused by the thermocouple track’s *R*, added to $V_{T}$. The measured respective voltages, for the applied positive ($I^{+}$) and negative ($I^{-}$) electrical currents, will therefore be $V^{+}=I^{+}R+V_{T}$ (step 1) and $V^{-}=I^{-}R+V_{T}$ (step 2), as shown in Figure 2. When added, the voltage due to the electrical resistance (created by the opposing current flows) cancels out, resulting in $V_{T}=\left(V^{+}+V^{-}\right)/2$ (step 3). The $V_{T}$ generated by an adjacent pixel is then directly measured and finally the crosstalk is calculated as the ratio between the two. Three chips were tested to check consistency, with the results presented in Table 1 (measurements with current source), showing ∼2.69% crosstalk. These values are comparable to current state-of-the-art thermopile FPAs [6,13,14], with our novel method being significantly simpler to apply. A comparison with the simulation results, also shown in Table 1, is discussed in the next section.

## 4. Numerical Simulations

**Figure 3:**
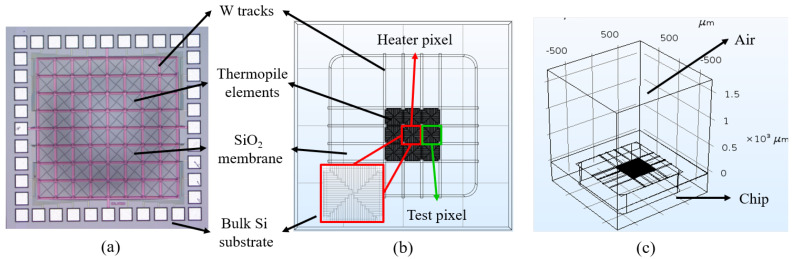
(**a**) Optical image of the thermopile array. Numerical model (**b**) top view and (**c**) 3D view.

To better understand the thermal behaviour of our device, we perform numerical simulations based on a finite elements method (FEM) model. For a comprehensive depiction of the thermopile’s behaviour, we use the heat transfer module, and the electric current module of the commercial software package COMSOL Multiphysics [27]. We reduce the complexity of our model by making the following two simplifications (a comparison between the structure of the real chip and that of our model is shown in Figure 3): we remove the metal pin pads around the membrane (Figure 3a,b), considering their negligible effect on both the electrical and thermal behaviour of the chip [20]; and, we simulate only 9 pixels at the centre of the membrane, and the metal tracks surrounding them (Figure 3b), considering the device contains 64 identical thermopiles (thus reducing the computation time). The 3-dimensional (3D) view of our model (Figure 3c), shows an air cube placed on top of the chip to account for heat losses in the air.

**Figure 4:**
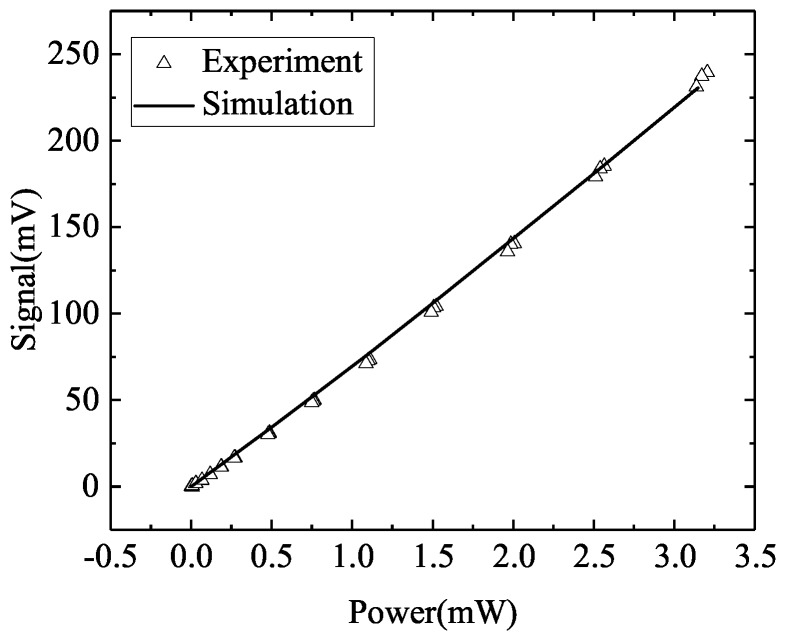
Comparison between simulated and experimentally generated thermoelectric voltages, by a heated pixel at electrical powers from 0 to 3.5 mW.

**Figure 5:**
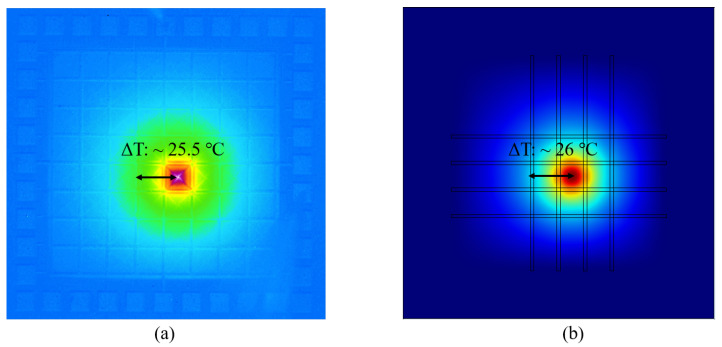
(**a**) Infrared image of the thermopile detector array chip measured using an IR thermal microscope. (**b**) Temperature distribution across the chip obtained from the simulations.

We compare the simulation and experimental results in Figure 4, showing good agreement for $V_{T}$ generated by a pixel as a function of input power. The corresponding crosstalks are 2.7% and 2.69% respectively for the simulations and experiments. We also compare the simulated temperature distribution (ΔT) across the heated pixel, to that measured using an IR thermal microscope, both obtained with a heating I∼ 200 μA (Figure 5). The respective 26 ${}^{\circ }$C (simulations) and 25.5 ${}^{\circ }$C (experiment) ΔTs show good agreement. It is expected that *T*-induced changes in the thermal properties of the materials, which would cause any distortion [16], will be limited here as *T* changes due to electrical heating are limited.

**Figure 6:**
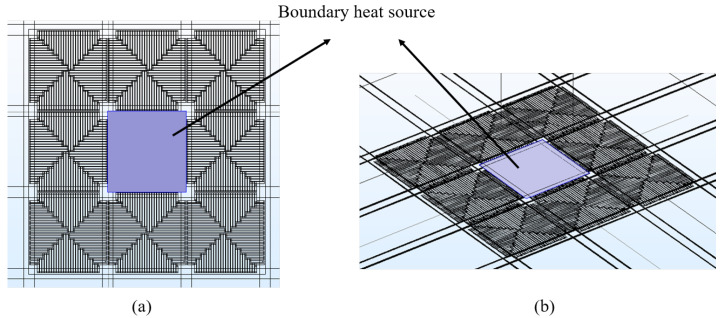
(**a**) Top view of the boundary heat source area. (**b**) 3D view of the boundary heat source area.

We then consider the effect of a uniform heat source across the pixel, as shown in Figure 6. We thus replace the current biasing with a boundary heat source, across the pixel, to define a constant power dissipation per unit area (a scenario mimicking a laser source illuminating a single pixel). In this case, the pixel is solely heated by the uniform boundary heat source (no current being applied); with the heating power being equivalent to that for the Joule heating scenario. Table 1 shows a comparison of simulated crosstalk values obtained for both heating approaches. The pixel’s responsivity, defined as the change in voltage response due to incident optical power [16], is also included.

The crosstalk simulated with the uniform heat source (3.02%) is slightly higher than that simulated with Joule heating (2.7%), while the responsivity shows an opposite trend, i.e., 13% lower. This is expected, as a non-uniform heat distribution across the pixel enhances ΔT, while inter-pixel heat diffusion is limited by the localised thermopile tracks.

## 5. Conclusions

In conclusion, we report a bi-directional electrical biasing method for crosstalk measurements of a thermopile-based IR detector array fabricated on a single dielectric membrane. The use of the thermopile itself as a heater, significantly simplifies the experimental setup, compared to the use of a laser source. We propose a theoretical model to further investigate crosstalk effects in these devices, which shows good agreement with the experimental results. Our results open new perspectives for these novel thermal devices.

## Figures and Tables

**Table 1:** Comparison between experimental and simulation results. Numerical simulations were implemented using both a current source and an uniform heat source across the thermopile elements.

Results	Pixel Resistance (kΩ)	Crosstalk (%)	Responsivity (V/W)
Measurements with current source	76.07	2.69	73.10
Simulations with current source	76.21	2.70	72.76
Simulations with uniform power source	76.21	3.02	63.05

## References

[1] Kimata M. (2017). Uncooled infrared focal plane arrays. IEEJ Trans. Electr. Electron. Eng..

[2] Rogalski A. (2012). Progress in focal plane array technologies. Prog. Quantum Electron..

[3] Popa D., Udrea F. (2019). Towards Integrated Mid-Infrared Gas Sensors. Sensors.

[4] Kruse P. (2011). Uncooled Thermal Imaging: Arrays, Systems, and Applications.

[5] Kimata M. Trends in small-format infrared array sensors. Proceedings of the 2013 IEEE SENSORS.

[6] Schaufelbuhl A., Schneeberger N., Munch U., Waelti M., Paul O., Brand O., Baltes H., Menolfi C., Huang Q., Doering E. (2001). Uncooled low-cost thermal imager based on micromachined CMOS integrated sensor array. J. Microelectromech. Syst..

[7] Holden A.J., Andresen B.F., Fulop G.F., Hanson C.M., Norton P.R., Robert P. (2013). Pyroelectric sensor arrays for detection and thermal imaging. Infrared Technology and Applications XXXIX.

[8] Minoli D., Sohraby K., Occhiogrosso B. (2017). IoT Considerations, Requirements, and Architectures for Smart Buildings—Energy Optimization and Next-Generation Building Management Systems. IEEE Internet Things J..

[9] Meijer G.C.M., Makinwa K.A.A., Pertijs M.A.P. (2014). Smart Sensor Systems: Emerging Technologies and Applications.

[10] Liang Q., Yu L., Zhai X., Wan Z., Nie H. Activity Recognition Based on Thermopile Imaging Array Sensor. Proceedings of the 2018 IEEE International Conference on Electro/Information Technology (EIT).

[11] John V., Mita S., Liu Z., Qi B. Pedestrian detection in thermal images using adaptive fuzzy C-means clustering and convolutional neural networks. Proceedings of the 2015 14th IAPR International Conference on Machine Vision Applications (MVA).

[12] Vieider C., Wissmar S., Ericsson P., Halldin U., Niklaus F., Stemme G., Källhammer J.E., Pettersson H., Eriksson D., Jakobsen H., Andresen B.F., Fulop G.F., Norton P.R. (2007). Low-cost far infrared bolometer camera for automotive use. Infrared Technology and Applications XXXIII.

[13] Popa D., Ali S.Z., Hopper R., Dai Y., Udrea F. (2019). Smart CMOS mid-infrared sensor array. Opt. Lett..

[14] Hopper R., Ali S.Z., Boual S., Luca A.D., Dai Y., Popa D., Udrea F. (2018). A CMOS-Based Thermopile Array Fabricated on a Single SiO2 Membrane. Proceedings.

[15] Hirota M., Nakajima Y., Saito M., Satou F., Uchiyama M., Andresen B.F., Fulop G.F., Strojnik M. (2003). 120 × 90 element thermopile array fabricated with CMOS technology. Infrared Technology and Applications XXVIII.

[16] Rogalski A. (2010). Infrared Detectors.

[17] Herwaarden A.V., Sarro P. (1986). Thermal sensors based on the seebeck effect. Sens. Actuators.

[18] Rogalski A. (2003). Infrared detectors: Status and trends. Prog. Quantum Electron..

[19] Wu H., Grabarnik S., Emadi A., de Graaf G., Wolffenbuttel R.F. (2009). Characterization of thermal cross-talk in a MEMS-based thermopile detector array. J. Micromech. Microeng..

[20] Falco C., Udrea F. On the application of a numerical model to improve the accuracy of the seebeck coefficient in CMOS materials. Proceedings of the 2017 IEEE SENSORS.

[21] Graf A., Arndt M., Sauer M., Gerlach G. (2007). Review of micromachined thermopiles for infrared detection. Meas. Sci. Technol..

[22] Hopper R., Ali S., Chowdhury M., Boual S., Luca A.D., Gardner J., Udrea F. (2014). A CMOS-MEMS Thermopile with an Integrated Temperature Sensing Diode for Mid-IR Thermometry. Procedia Eng..

[23] Hirakawa K. Cross-talk explained. Proceedings of the 2008 15th IEEE International Conference on Image Processing.

[24] Agranov G., Berezin V., Tsai R.H. (2003). Crosstalk and microlens study in a color CMOS image sensor. IEEE Trans. Electron Devices.

[25] Udrea F., De Luca A. CMOS technology platform for ubiquitous microsensors. Proceedings of the 2017 International Semiconductor Conference (CAS).

[26] DiSalvo F.J. (1999). Thermoelectric Cooling and Power Generation. Science.

[27] Yushanov S.P., Gritter L.T., Crompton J.S., Koppenhoefer K.C. Multiphysics analysis of thermoelectric phenomena. Proceedings of the 2011 COMSOL Conference.

